# Geomorphological evidence of large vertebrates interacting with the seafloor at abyssal depths in a region designated for deep-sea mining

**DOI:** 10.1098/rsos.180286

**Published:** 2018-08-22

**Authors:** Leigh Marsh, Veerle A. I. Huvenne, Daniel O. B. Jones

**Affiliations:** 1 National Oceanography Centre, European Way, Southampton SO14 3ZH, UK; 2 Ocean and Earth Science, University of Southampton, Waterfront Campus, Southampton SO14 3ZH, UK

**Keywords:** marine mammals, deep-sea mining, autonomous underwater vehicle, deep-diving mammals, Clarion–Clipperton zone, ichnology

## Abstract

Exploration licences for seafloor mineral deposits have been granted across large areas of the world's oceans, with the abyssal Pacific Ocean being the primary target for polymetallic nodules—a potentially valuable source of minerals. These nodule-bearing areas support a large diversity of deep-sea life and although studies have begun to characterize the benthic fauna within the region, the ecological interactions between large bathypelagic vertebrates of the open ocean and the abyssal seafloor remain largely unknown. Here we report seafloor geomorphological alterations observed by an autonomous underwater vehicle that suggest large vertebrates could have interacted with the seafloor to a maximum depth of 4258 m in the recent geological past. Patterns of disturbance on the seafloor are broadly comparable to those recorded in other regions of the world's oceans attributed to beaked whales. These observations have important implications for baseline ecological assessments and the environmental management of potential future mining activities within this region of the Pacific.

## 1. Introduction

The abyssal seafloor represents approximately 85% of the global seafloor [1], yet many of the ecosystems and species that it sustains are largely unknown because of the difficulties in studying such a vast and remote environment. Advances in deep-submergence technologies have allowed abyssal research to be conducted at spatially confined environments such as hydrothermal vents [2], trenches [3] and submarine canyons [4]. However, studies at the scale necessary to understand the ecology and importance of sediment-hosted abyssal plains are still rare [5].

**Figure 1.**
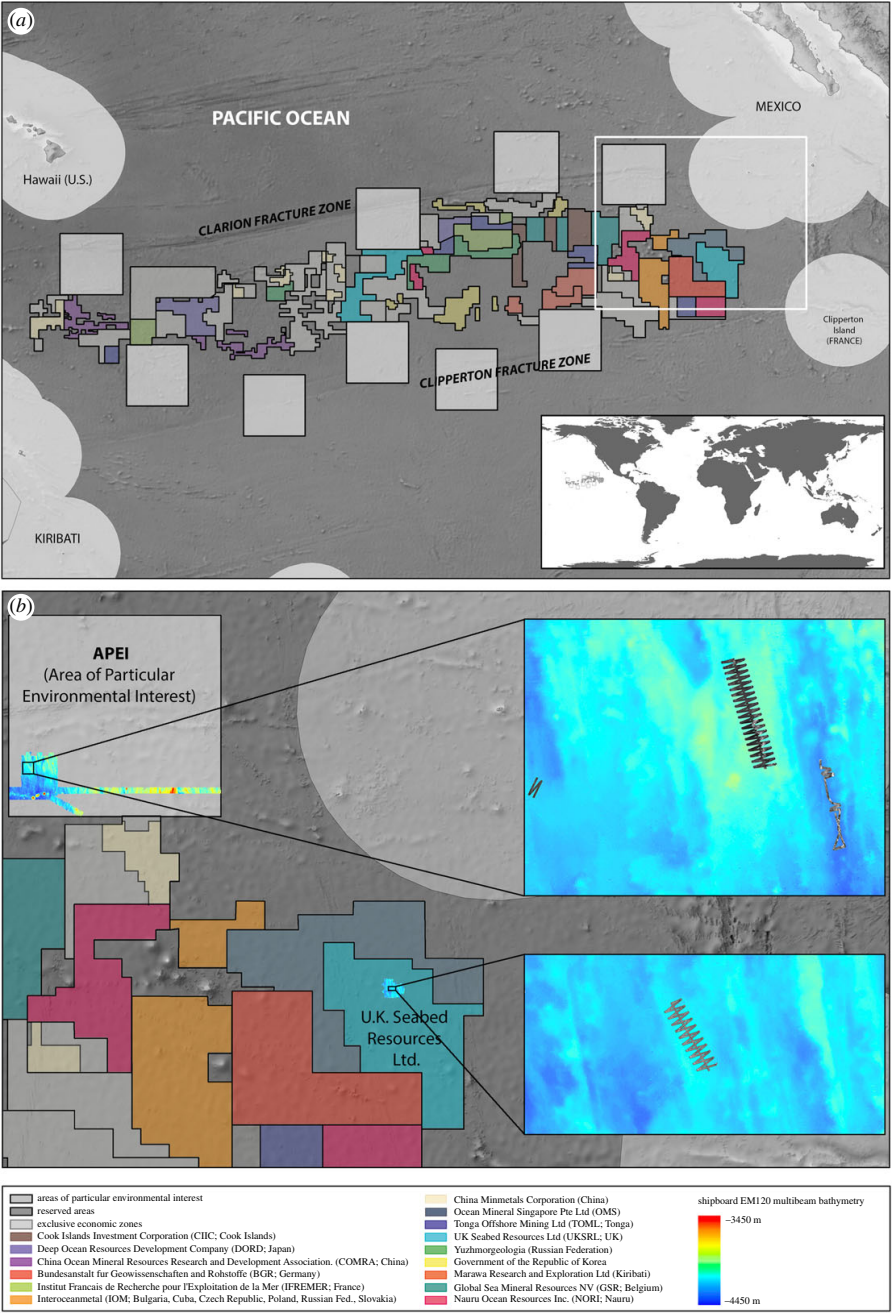
(*a*) Region targeted for polymetallic nodule mining in the Clarion–Clipperton Zone (CCZ), Pacific Ocean. Exploration claims are delineated by coloured boxes. The Areas of Particular Environmental Interest (APEI) are shown in grey. (*b*) During expedition JC120, parts of the northeasternmost APEI and the UK claim zone were surveyed. (Inset top) EM120 shipboard multibeam from the APEI with *Autosub6000* M79, M81 and M83 side-scan sonar missions. (Inset bottom) EM120 shipboard multibeam from the UK claim zone with *Autosub6000* M85 side-scan sonar mission.

The Clarion–Clipperton Zone (CCZ) in the Northeast Pacific covers around 6 million km^2^ and ranges 3000–6000 m in depth [6]. This region has attracted significant interest over the past decade owing to the presence of polymetallic nodules—a targeted mineral resource of cobalt, copper and rare earth elements in the deep sea. The International Seabed Authority (ISA) is the organization established by the 1982 UN Convention on the Law of the Sea (UNCLOS) to manage seabed mining beyond the areas of national jurisdiction (ABNJ) and, as of January 2018, the ISA had granted 16 exploration contracts within the CCZ (figure 1).

It is widely accepted that nodules provide a home for a wide variety of suspension feeders and specialized invertebrate megafauna, which are dependent on the hard substratum provided by the nodules in an otherwise sediment-dominated environment [7]. To quantify the ecological importance of these areas, under their contractual arrangements with the ISA, exploration contractors are obliged to undertake environmental baseline biological studies. Researchers have begun to understand the structure of benthic faunal assemblages in the CCZ [7,8]; however, the ecological interactions between bathypelagic vertebrates of the open ocean and the abyssal seafloor remain largely unknown. Therefore, serendipitous observations during industry-led deep-submergence work can be of significant interest [9].

This paper suggests that large vertebrates have used the abyssal seafloor in the CCZ in the recent geological past. We demonstrate that sequential depressions represented by acoustic shadows from autonomous underwater vehicle (AUV) geophysical surveys observed in the CCZ are spatially comparable and, from limited seafloor imagery, represent a morphology akin to those inferred from beaked whales in the Atlantic [10] and Mediterranean [11,12].

## 2. Material and methods

Managing Impacts of Deep-seA reSource exploitation (MIDAS) is an EU-funded project aimed at building the knowledge base to underpin sound environmental policies in relation to deep-sea mining. As part of this project, the RRS *James Cook* visited the CCZ in April to May 2015 (expedition JC120; [13]), focusing on the UK Seabed Resources Ltd Claim Zone and the northeasternmost Area of Particular Environmental Interest (APEI) defined by the ISA [14]. This expedition used the *Autosub6000* AUV [15] along with a suite of other data collection methods to form an environmental baseline for this area.

Operations were constrained within an approximately 5500 km^2^ area of seafloor within the APEI and within approximately 1100 km^2^ of the UK Seabed Resources Ltd Claim Zone. Shipboard EM120 multibeam echosounder data acquired and gridded at 100 m resolution were used to create bathymetric derivatives for survey planning. In the bathymetric data, several morphological features were clearly visible in the region. To try and capture this variation, a stratified random survey was designed using objective criteria [13]. High-resolution acoustic mapping data (multibeam echosounder and side-scan sonar data) from defined strata were recorded using *Autosub6000* (figure 1).

**Figure 2.**
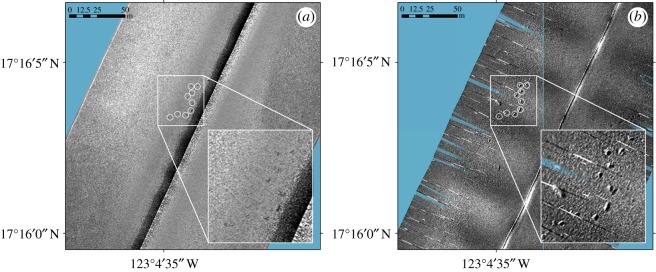
Detail of independently obtained high-frequency side-scan sonar at (*a*) 15 m (traces faint) and (*b*) 3 m (easily resolved) altitude.

*Autosub6000* is equipped with an Edgetech FS2200-M dual-frequency side-scan sonar and sub-bottom profiler [16]. The high-frequency setting (410 kHz) of the Edgetech side-scan sonar was used both for short dedicated transects (15 m altitude) and during photo-transects (3 m altitude) carried out by the AUV. The extremely low incidence angles at approximately 3 m altitude allowed the sonar to image very shallow depressions (represented as acoustic shadows), which could also be seen faintly in the 15 m altitude data (figure 2). However, the depressions were not visible in lower-frequency, or higher-altitude data.

In total, four *Autosub6000* missions (M79, M81, M83 within the APEI and M85 within the UK claim zone) were achieved at the optimal altitude (3 m) and frequency (410 kHz) to allow seafloor depressions to be resolved. Processing of the high-frequency side-scan sonar data was completed using the NOC-developed PRISM software package [17]. Results were collated in ERDAS Imagine and compiled into a single image mosaic. All resolvable depressions were digitized in ArcGIS 10.3 as a point file. From this shapefile, a series of ‘tracks’ (curvilinear strings of sequential depressions) were selected for further analysis. As the detection of depressions varies with the quality of the side-scan data, not all depressions were easily resolved. Therefore, objective criteria were designed to assess the spatial patterns of the depressions within a given track. For M83 and M85, only 1 and 2 tracks were detected, respectively. Both of these sets of tracks had a minimum number of 6 depressions (i.e. 5 mid-point to mid-point distances). As a result, 6 was set as the minimum number of sequential, detectable depressions for M79 and M80. Additionally, the tracks were not counted if they crossed the nadir of the geophysical survey (the centre region of the side-scan sonar swath, which represents the seafloor directly under the sonar, and tends to be poorly resolved as a result of the geometry of the acoustic signal). If a track crosses the nadir, a depression may not have been detected in the region of the seafloor in which the nadir occurs, which would result in an incorrect distance between depressions being calculated. Depression length and width were measured directly from the raw side-scan data using the Edgetech DISCOVER 4200 software, and the distance between consecutive depressions within a given track was determined using the analysis toolbox in ArcGIS 10.3. For comparison, the distance between depressions was also calculated from the high-resolution photomosaic published in [12].

Seabed imagery was successfully collected in a zig-zag survey design randomly located within the acoustic survey areas of M79, M81 and M83 within the APEI. Photographic data were obtained using two Point Gray Research Inc. Grasshopper 2 cameras on the AUV, one mounted vertically and the other obliquely looking forward [5]. The field of view from the vertically mounted camera was approximately 1.7 m^2^. AUV photography and high-frequency side-scan surveys were acquired simultaneously at a 3 m altitude. As a result, the photographs provided by the vertically mounted camera run through the nadir (approx. 1.5 m width) of the geophysical data, preventing simultaneous assessment of features in both the photographs and side-scan data.

Seabed photographs from the successful AUV photography missions were reviewed. Owing to the perpendicular angle of the camera to the seafloor of the vertically mounted camera, any depressions or relief in the seafloor topography is difficult to resolve. Only limited occurrences of the depressions were observed in the forward-facing camera, and no laser scaling is provided in the oblique view images. Therefore, no further morphometric data could be obtained.

## 3. Results and discussion

**Figure 3.**
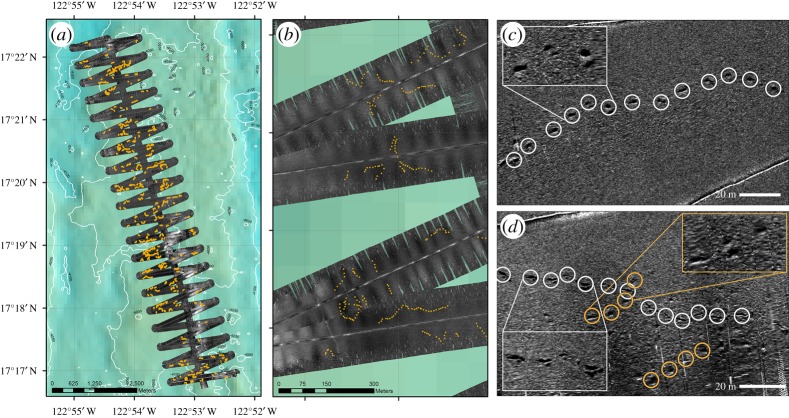
*Autosub6000* Mission 81 (M81) within APEI. (*a*) High-frequency (410 kHz) side-scan sonar acquired at 3 m altitude. Areas with high acoustic backscatter are represented in light grey, low acoustic backscatter in dark grey. Orange circles indicate depressions that have been digitized in ArcGIS 10.3. (*b*) Zoom of M81 indicating sequential depressions or ‘tracks’. (*c*) Single sequence of depressions (track) from M81. Depth: 4023 m. (*d*) Overlapping tracks of differing ages. White tracks show high contrast and sharp edges indicating relatively younger tracks than those in orange with lower contrast and less definitive edges. Depth: 4041 m.

**Figure 4.**
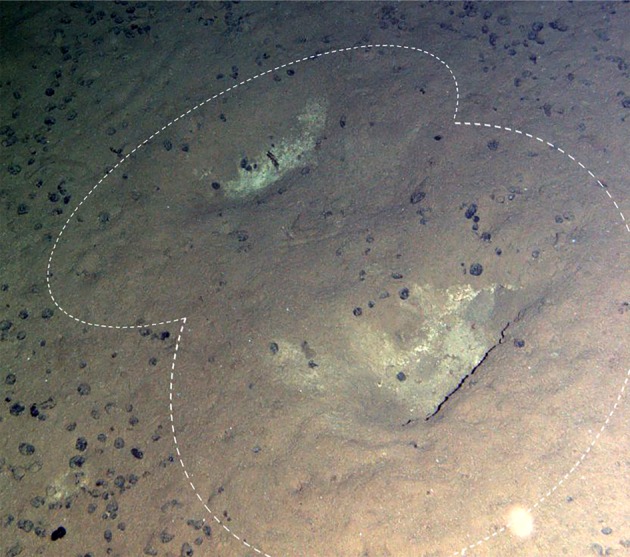
Image provided from the oblique camera from *Autosub6000* Mission 79 (M79) within the APEI shows two depressions, inferred to be those also observed from acoustic data. White dashed line indicates an area where sediment from the excavation has subsequently covered nodules within the vicinity. Eroded edges would suggest that these particular depressions have not been made in recent years. Depth: 4153 m.

AUV acoustic seabed surveys of an area within the Clarion–Clipperton Zone (CCZ; figure 1) revealed elongated depressions across the seafloor fabric (figure 3). A total of 3539 depressions were counted over side-scan sonar data covering 21.8 km^2^ at water depths from 3999 to 4258 m in the northeastern CCZ (table 1). These depressions formed curvilinear tracks along the seafloor, consisting of up to 21 depressions spaced between 6 and 13 m apart. The seafloor depressions followed variable paths, with distinct tracks spaced irregularly over much of the area surveyed and occasionally crossing (figure 3). Depressions consisted of irregular furrows on the seafloor (mean 0.97 m wide and 2.57 m long) approximately 0.13 m deep (data provided from figure 3). Limited observations of individual depressions were also visible on seafloor imagery (figure 4), with these observations broadly corresponding in morphology to those inferred from the side-scan data.

**Table 1.** Summary of data on geomorphic alterations of the seafloor attributed to whales from high-frequency AUV side scan (this study) and ROV photomosaic from Roman *et al*. [12]. Mean distance between depressions measured from the centre point of each depression. The deepest observation is indicated in italics.

vehicle	data type	location	depth range (m)	area covered (km^2^)	total resolvable depressions	density (km^−2^)	number of tracks (greater than 6 sequential depressions)	mean distance between depressions (±1 s.d.)	reference
**AUV (M79)**	side scan	APEI CCZ, Pacific	4195–4160	0.859	512	596	23	8.14 ± 2.83	this study
**AUV (M81)**	side scan	APEI CCZ, Pacific	4117–3999	11.277	2951	262	30	8.78 ± 2.88	this study
**AUV (M83)**	side scan	APEI CCZ, Pacific	*4258–*4227	3.223	34	11	1	13.39 ± 1.19	this study
**AUV (M85)**	side scan	UK Claim CCZ, Pacific	4120–4111	6.490	42	3	2	6.44 ± 1.26	this study
**ROV**	photomosaic	Seamount, Mediterranean	1000–800	0.00116	17 (identified within publication)	14 655^a^	1	8.09 ± 1.51	Roman *et al.* [12]

^a^Probably an overestimate owing to targeted sampling using ROV.

The CCZ has an extremely low food supply (particulate organic carbon flux approximately 1 gC m^−2^ y^−1^; [18]), bottom currents (1–9 cm s^−1^) [19], sedimentation (0.35 cm kyr^−1^) [20] and bioturbation rates (3–6 cm^2^ yr^−1^) [21], suggesting tracks may be preserved for long periods of time. Based on the sedimentation rate alone, a maximum age for these tracks in the CCZ can be estimated, with it taking approximately 28 kyr to fill a typical trace depression (0.1 m deep). The geophysical data presented here appear to show tracks of various ages based on their acoustic shadows; shadows with sharp edges are inferred to be from more recent depressions, while shadows with lower reflective contrast are inferred to correspond to older depressions, having experienced infilling by sedimentation, bioturbation and erosion by bottom currents (figure 3).

There is no direct evidence for the cause of the depressions. No known geological mechanism exists for the formation of curvilinear sequences of shallow depressions in deep-water low-permeability sediments with no advective seabed fluid flow expected [22]. The size and frequency of depressions suggests that only a large organism could be responsible. The largest fish species (less than 1.02 m) known to inhabit these water depths in the Pacific are *Coryphaenoides armatus* and *Coryphaenoides yaquinae* [23]. These species of abyssal fish have reduced locomotory capacity [24] and slow swimming speeds (less than 0.15 m s^−1^) [25] and are unlikely to be able to create relatively deep, sequential depressions in clay sediments [20] several times longer than their body lengths. Complex behaviours associated with nesting [26] have not been observed in deep-sea fishes and would be energetically extremely costly to make in this environment.

Geomorphic alterations of the seafloor caused by marine tetrapods have been recognized in both modern [27] and palaeontological records [28]. In modern oceans, these seabed alterations (e.g. gouges, pits, tracks, etc.) have been well documented from narwhals and beluga whales in fjords [29], to walruses and humpback whales on the shallow continental shelf [30]. The characteristic patterns observed within this study and the distance between the midpoints of consecutive depressions within a given track are similar to seafloor modifications identified from remotely operated vehicle (ROV) video in the Mediterranean (separation distance 5–10 m [9]; separation distance 6–10 m [12]) with their occurrence being attributed to foraging beaked whales. From limited imagery, the depressions are also of similar morphology to those presented in previous studies [9,10]. However, it is important to note that some inconsistencies are observed, specifically when compared with those from Woodside *et al*. [9], where a narrow central groove is observed superimposed on a larger seafloor depression. These differences could be attributed to either (a) the methodologies obtaining the size and morphology of depression—side-scan sonar can be used only to provide approximate measurements based on acoustic shadows and may not resolve subtleties in the morphology (i.e. a groove feature within a depression), while measurements from oblique ROV videography can again only provide an estimate of size, but will give greater visual resolution; (b) the relative age of the depression, which may result in altered morphology (owing to seafloor processes); and/or finally; (c) different species being responsible for making the depressions.

Despite being the most speciose family of the cetaceans, deep-diving beaked whales of the family Ziphiidae represent the most elusive whales in the world's oceans, with species new to science still being discovered [31]. Unlike shallow-water counterparts (e.g. Delphinidae), or large filter-feeding relatives (e.g. Balaenidae), deep-diving whales are challenging to study owing to their open-ocean pelagic nature, small fin with a low-surface profile and inconspicuous surface blows [32]. To date, five extant species of beaked whale (Ziphiidae) and the deep-diving sperm whale (*Physeter microcephalus*) are likely to occur in the waters of the Pacific Ocean within the CCZ region [33]. While it is not possible to identify which species (extinct or extant) could be responsible, our observations of seafloor modifications within the 4258 m contour exceed the deepest known dive [34] by any species of whale by over 1200 m.

Throughout the CCZ, there is a high incidence of fossil whale bones from the Family Ziphiidae [35]. Furthermore, a recent ‘whale-fall’ of a small odontocete has been observed at the 4142 m depth [36]. Although the presence of extinct fossilized whale bones and the observation of a recently deceased odontocete do not demonstrate that these animals were (or are) capable of diving to these abyssal depths, it does confirm their presence over geological timescales within the CCZ region. When we consider the maximum eustatic sea-level amplitude, we would suggest that even if these marks were made during the last glacial maximum, when water depths in the Pacific Ocean were 125–135 m lower [37], the species responsible would still have been capable of diving to depths of nearly 4000 m. Anatomical studies suggest that cranial air spaces in Cuvier's beaked whales could withstand a dive to depths of 5000 m [38], and although the physiological limits of diving are unknown, it is conceivable that a whale capable of diving to these depths exists in our oceans today.

Several hypotheses have been proposed as to why whales may cause such indentations on the seafloor. These include (i) removing parasites or dead skin [11], behaviour that is known from other odontocetes in shallow water [39–41]; (ii) foraging in the sediments for prey items (benthic or infaunal invertebrates) [9]; or (iii) trying to catch motile bentho-pelagic species such as cephalopods and fish [10]. As a result of (ii) and (iii), it has been suggested that individuals may be ingesting debris accidently [42]. However, there are examples where other marine tetrapods are thought to (iv) intentionally ingest coarse material to regulate buoyancy [43].

The characteristic curvilinear pattern observed here would suggest that an individual would come into contact with the seafloor multiple times during one dive. Therefore, it appears that the individual is actively excavating the sediment. Invertebrate benthic biomass in abyssal plains is reported to be low (approx. 4 g m^−2^) [44]—unlike the large shallow-water feeding mysticetes on the continental shelf (which filter-feed on sediments containing approx. 170 g m^−2^ of ampeliscid amphipods) [30], this bentho-abyssal approach for a species of whale would represent an energetically costly mode of foraging for such parsimonious feeding.

Some species of beaked whale are known to feed in close proximity to the seafloor (*Mesoplodon densirostris*) [45], while other species (*Ziphius cavirostris*) in the east [46,47] and west North Pacific (*Berardius bairdii*) [48] are reported to feed on abyssal bentho-pelagic fish, including the Macrouridae (or grenadiers). Maximum abundance of abyssal fish (including grenadiers) has recently been estimated at 723 individuals km^−2^ [49], which represents a significant food resource at depths beyond 4000 m. Although an efficient predatory method of echolocation [50] and suction feeding is employed by beaked whales [51] and other known species of odontocetes, this does not preclude a chase after escaping prey. Energetic foraging has been shown in the echolocating, suction-feeding, short-finned pilot whale [52], therefore it is plausible that sequential tracks could be a by-product of whale chasing prey [10].

Ingesting material (including nodules) for ballast—a hypothesis first postulated following the *Challenger* expedition [35]*—*is documented in both groups of fossil (with gastrolith function reviewed by [53]) and extant families of marine tetrapods [43,54,55] with the primary role inferred to regulate buoyancy in species that ‘fly’ or ‘glide’ underwater using hydrofoil fins (e.g. ottarids, penguins, pleisosaurs). To date, gastroliths (or ‘stomach stones’) have not been considered to play a major role in cetaceans that swim primarily using a caudal fin [43]. However, research suggests prolonged periods of ‘gliding’ are a behavioural response by caudal-fin swimming marine mammals to improve energetic efficiency during deep dives [56] and that buoyancy [57,58] and biomechanical strategies [59] influence these different swimming gaits. Both physiological [60,61] and behavioural adaptions [59] would suggest that deep-diving species have the capability to forage at depth without the need to ingest large quantities of sediment or stone to add ballast. However, gastroliths have been documented in both individuals of Baird's beaked whale (*B. bairdii*) [48] and the sperm whale (*P. microcephalus)* [62], which has recently been reported to follow the seafloor in deep ‘benthic’ dives [63] and, from historic observations, even ‘plough’ the seafloor [64]. As to whether the occurrences of gastroliths in these species can be attributed to accidental ingestion [42] or individuals actively partaking in some form of geophagy remains unknown.

Although with the dataset available we cannot determine which species is responsible, or why they are creating these disturbances on the seafloor, the precautionary principle must be adhered to. Sperm whales and all the extant species of Ziphiidae are likely to occur within the CCZ and research would suggest that some of these deep divers may be capable of using the sea floor within this region; this may have important implications for management of existing and planned marine industrial activities. All of these species are on the IUCN Red List of Threatened Species (http://www.iucnredlist.org/, accessed 2018) and Article 120 of the 1982 UNCLOS puts in place measures for their conservation.

Monitoring of marine mammals in areas of industrial activity will be important, and current guidance from the International Seabed Authority (ISBA/19/LTC/8) requires contractors to record sightings of marine mammals to ascertain spatial and temporal variability of species within the region. For deep-diving whales that are renowned for their elusive lifestyle and sometimes inconspicuous identification at the surface, traditional vessel-based marine mammal observations may not be effective [32] and active management to avoid impacts to whales from underwater noise, to which they are particularly sensitive, will be necessary.

Whichever taxa may be responsible for these sea-floor interactions, this study highlights how the use of ultra-low altitude deep-submergence AUVs will become invaluable in detecting these observations over large scales (kilometres) and deriving seafloor habitat utilization maps, while human-directed ROV observations will be key in visually examining and sampling these disturbances further. Deep-diving whales can be found throughout our global oceans—to what extent they are using and altering the seafloor environment remains unknown. The observations presented in this study highlight the number of important discoveries still to be made in our deep ocean and, yet, we are already looking to exploit a habitat that we know very little about.

## Supplementary Material

LMarsh_Track_Distance_ESM

## Data accessibility

The datasets supporting this article have been uploaded as part of the electronic supplementary material.

## References

[1] Harris PT, Macmillan-Lawler M, Rupp J, Baker EK 2014 Geomorphology of the oceans. Mar. Geol. 352, 4–24. (10.1016/j.margeo.2014.01.011)

[2] Connelly DP et al. 2012 Hydrothermal vent fields and chemosynthetic biota on the world's deepest seafloor spreading centre. Nat. Commun. 3, 620 (10.1038/ncomms1636) 22233630 PMC3274706

[3] Bowen AD et al. 2009 The Nereus hybrid underwater robotic vehicle. Underwater Technol. 28, 79–89. (10.3723/ut.28.079)

[4] Huvenne VAI, McPhail SD, Wynn RB, Furlong M, Stevenson P 2009 Mapping giant scours in the deep ocean. Eos, Trans. Am. Geophys. Union 90, 274–275. (10.1029/2009EO320002)

[5] Morris KJ et al. 2014 A new method for ecological surveying of the abyss using autonomous underwater vehicle photography. Limnol. Oceanogr. Methods 12, 795–809. (10.4319/lom.2014.12.795)

[6] Wedding LM et al. 2015 Managing mining of the deep seabed. Science 349, 144–145. (10.1126/science.aac6647) 26160934

[7] Vanreusel A, Hilario A, Ribeiro PA, Menot L 2016 Threatened by mining, polymetallic nodules are required to preserve abyssal epifauna. Sci. Rep. 6, 26808 (10.1038/srep26808) 27245847 PMC4887785

[8] Amon DJ et al. 2016 Insights into the abundance and diversity of abyssal megafauna in a polymetallic-nodule region in the eastern Clarion-Clipperton Zone. Sci. Rep. 6, 30492 (10.1038/srep30492) 27470484 PMC4965819

[9] Woodside JM, David L, Frantzis A, Hooker SK 2006 Gouge marks on deep-sea mud volcanoes in the eastern Mediterranean: Caused by Cuvier's beaked whales? Deep-Sea Res. Part Oceanogr. Res. Pap. 53, 1762–1771. (10.1016/j.dsr.2006.08.011)

[10] Auster PJ, Watling L 2009 Beaked whale foraging areas inferred by gouges in the seafloor. Mar. Mammal. Sci. 26, 226–233. (10.1111/j.1748-7692.2009.00325.x)

[11] Bell RJ, Mayer L, Konnaris K, Bell KLC, Ballard R 2011 Potential marine mammal induced seafloor scours on Eratosthenes Seamount. In New frontiers in ocean exploration: the E/V Nautilus 2010 field season oceanography (eds Bell KLC, Fuller S), Oceanography 24 (supplement), 30. (10.5670/oceanog.24.1.supplement)

[12] Roman C et al. 2012 New frontiers in ocean exploration: the E/V Nautilus 2011 field season oceanography. In New Frontiers in ocean exploration: the E/V Nautilus 2011 field season oceanography (eds Bell KLC, Elliott KP, Martinez C, Fuller S), Oceanography 24 (supplement), 42–45. (10.5670/oceanog.24.1.supplement)

[13] Jones DOB. 2015 National Oceanography Centre Cruise Report No. 32 RRS James Cook Cruise JC120.

[14] Lodge M, Johnson D, Le Gurun G, Wengler M, Weaver P, Gunn V 2014 Seabed mining: International Seabed Authority environmental management plan for the Clarion-Clipperton Zone: a partnership approach. Mar. Policy 49, 66–72. (10.1016/j.marpol.2014.04.006)

[15] McPhail S, Furlong M, Pebody M 2010 Low-altitude terrain following and collision avoidance in a flight-class autonomous underwater vehicle. J. Eng. Marit. Environ. 224, 279–292. (10.1130/G39091.1)

[16] Wynn RB et al. 2014 Autonomous underwater vehicles (AUVs): their past, present and future contributions to the advancement of marine geoscience. Mar. Geol. 352, 451–468. (10.1016/j.margeo.2014.03.012)

[17] Le Bas TP, Huvenne VAI 2009 Acquisition and processing of backscatter data for habitat mapping: comparison of multibeam and sidescan systems. Appl. Acoust. 70, 1248–1257. (10.1016/j.apacoust.2008.07.010)

[18] Lutz MJ, Caldeira K, Dunbar RB, Behrenfeld MJ 2007 Seasonal rhythms of net primary production and particulate organic carbon flux to depth describe the efficiency of biological pump in the global ocean. J. Geophys. Res. Oceans 112, C10011 (10.1029/2006JC003706)

[19] Hayes SP 1979 Benthic current observations at DOMES sites A, B, and C in the tropical North Pacific Ocean. In Marine geology and oceanography of the Pacific manganese nodule province (eds Bischoff JL, Piper DZ), pp. 83–112. Boston, MA: Springer.

[20] Mewes K, Mogollón JM, Picard A, Rühlemann C, Kuhn T 2014 Impact of depositional and biogeochemical processes on small scale variations in nodule abundance in the Clarion–Clipperton Fracture Zone. Deep-Sea Res. Part I 91, 125–141. (10.1016/j.dsr.2014.06.001)

[21] Mewes K et al. 2016 Diffusive transfer of oxygen from seamount basaltic crust into overlying sediments: an example from the Clarion–Clipperton Fracture Zone. Earth Planet. Sci. Lett. 433, 215–225. (10.1016/j.epsl.2015.10.028)

[22] Kuhn T et al. 2017 Widespread seawater circulation in 18–22 Ma oceanic crust: impact on heat flow and sediment geochemistry. Geology 45, 799–802.

[23] Hoff GR, Stevenson DE, Orr JW 2016 Guide to the gadiform fishes of the eastern North Pacific: cods, grenadiers, hakes, morids, codlings [Internet]. U.S. Department of Commerce, National Oceanic and Atmospheric Administration, National Marine Fisheries Service. Alaska Fisheries Science Center See https://repository.library.noaa.gov/view/noaa/5354.

[24] Drazen JC, Seibel BA 2007 Depth-related trends in metabolism of benthic and benthopelagic deep-sea fishes. Limnol. Oceanogr. 52, 2306–2316. (10.4319/lo.2007.52.5.2306)

[25] Priede IG, Smith KL, Armstrong JD 1990 Foraging behavior of abyssal grenadier fish: inferences from acoustic tagging and tracking in the North Pacific Ocean. Deep Sea Res. Part Oceanogr. Res. Pap. 37, 81–101. (10.1016/0198-0149(90)90030-Y)

[26] Kawase H, Okata Y, Ito K 2013 Role of huge geometric circular structures in the reproduction of a marine pufferfish. Sci. Rep. 3, 2106 (10.1038/srep02106) 23811799 PMC3696902

[27] Nelson CH, Johnson KR 1987 Whales and walruses as tillers of the sea floor. Sci. Am. 256, 112–117. (10.1038/scientificamerican0287-112)

[28] McHenry C, Cook AG, Wroe S 2005 Bottom-feeding plesiosaurs. Science 310, 75 (10.1126/science.1117241) 16210529

[29] Hein FJ, Syvitski JPM 1989 Sea floor gouges and pits in deep fjords, Baffin Island: possible mammalian feeding traces. Geo-Mar. Lett. 9, 91–94. (10.1007/BF02430429)

[30] Johnson KR, Nelson CH 1984 Side-Scan sonar assessment of gray whale feeding in the Bering Sea. Science. 225, 15–17. (10.1126/science.225.4667.1150) 17782421

[31] Morin PA et al. 2016 Genetic structure of the beaked whale genus *Berardius* in the North Pacific, with genetic evidence for a new species. Mar. Mammal Sci. 1–16.

[32] Barlow J, Gisner R 2006 Mitigating, monitoring and assessing the effects of anthropogenic sounds on beaked whales. J. Cetacean Res. Man. 7, 11.

[33] Halpin P et al. 2009 OBIS-SEAMAP: the world data center for marine mammal, sea bird, and sea turtle distributions. Oceanography 22, 104–115. (10.5670/oceanog.2009.42)

[34] Schorr GS, Falcone EA, Moretti DJ, Andrews RD 2014 First long-term behavioral records from Cuvier's beaked whales (*Ziphius cavirostris*) reveal record-breaking dives. PLoS ONE 9, e0092633 (10.1371/journal.pone.0092633) PMC3966784 24670984

[35] Murray J, Renard A 1891 Report on deep-sea deposits based on specimens collected during the voyage of HMS Challenger in the years 1872–1876. London, UK: HMSO.

[36] Amon DJ, Hilario A, Arbizu PM, Smith CR 2016 Observations of organic falls from the abyssal Clarion-Clipperton Zone in the tropical eastern Pacific Ocean. Mar. Biodivers. 47, 311 (10.1007/s12526-016-0572-4)

[37] Peltier WR, Fairbanks RG 2006 Global glacial ice volume and last glacial maximum duration from an extended Barbados sea level record. Quat. Sci. Rev. 25, 3322–3337. (10.1016/j.quascirev.2006.04.010)

[38] Cranford TEDW et al. 2008 Anatomic geometry of sound transmission and reception in Cuvier's beaked whale (*Ziphius cavirostris*). Anat. Rec. 378, 353–378. (10.1002/ar.20652) 18228579

[39] Smith TG, Aubin DJS, Hammill MO 1992 Rubbing behaviour of belugas, *Delphinapterus leucas*, in a high Arctic estuary. Can. J. Zool. 70, 2405–2409. (10.1139/z92-322)

[40] Fortune SME, Koski WR, Higdon JW, Trites AW, Baumgartner MF, Ferguson SH 2017 Evidence of molting and the function of ‘rock-nosing’ behavior in bowhead whales in the eastern Canadian Arctic. PLoS ONE 12, 1–15. (10.1371/journal.pone.0186156) PMC5699794 29166385

[41] Ford JKB 2006 *An assessment of critical habitats of resident killer whales off the Pacific coast of Canada*. Research document 2006/072, Fisheries and Oceans Canada.

[42] Walker WA, Coe JM 1989 Survey of marine debris ingestion by odontocete cetaceans. In Proc. 2nd Int. Conf. on Marine Debris, Honolulu, Hawaii, 2–7 April, pp. 747–774. NOAA.

[43] Taylor MA 1993 Stomach stones for feeding or buoyancy? The occurrence and function of gastroliths in marine tetrapods. Phil. Trans. R. Soc. Lond. B 341, 163–175. (10.1098/rstb.1993.0100)

[44] Durden JM, Bett BJ, Jones DOB, Huvenne VAI, Ruhl HA 2015 Abyssal hills: hidden source of increased habitat heterogeneity benthic megafaunal biomass and diversity in the deep sea. Prog. Oceanogr. 137, 209–218. (10.1016/j.pocean.2015.06.006)

[45] Arranz P, Aguilar de Soto N, Madsen PT, Brito A, Bordes F, Johnson MP 2011 Following a foraging fish-finder: diel habitat use of Blainville's beaked whales revealed by echolocation. PLoS ONE 6, e0028353 (10.1371/journal.pone.0028353) PMC3233560 22163295

[46] West KL, Walker WA, Baird RW, Mead JG, Collins PW 2017 Diet of Cuvier's beaked whales *Ziphius cavirostris* from the North Pacific and a comparison with their diet world-wide. Mar. Ecol. Pro. Ser. 574, 227–242. (10.3354/meps12214)

[47] Adams J, Walker WA, Burton EJ, Harvey JT 2015 Stomach contents of a Cuvier's beaked whale (*Ziphius cavirostris*) stranded in Monterey Bay, California. Northwest Nat. 96, 93–98. (10.1898/NWN14-10.1)

[48] Walker WA, Mead JG, Brownell RL 2002 Diets of Baird's beaked whales, *Berardius Bairdii*, in the Southern Sea of Okhotsk and off the Pacific coast of Honshu, Japan. Mar. Mammal Sci. 18, 902–919. (10.1111/j.1748-7692.2002.tb01081.x)

[49] Milligan RJ et al. 2016 High-resolution study of the spatial distributions of abyssal fishes by autonomous underwater vehicle. Sci. Rep. 6, 1–12. (10.1038/srep26095) 27180728 PMC4867640

[50] Johnson MP, Madsen PT, Zimmer WMX, Aguilar de Soto N, Tyack PL 2004 Beaked whales echolocate on prey. Proc. Biol. Sci. 271, S383–S386. 15801582 10.1098/rsbl.2004.0208 PMC1810096

[51] Heyning J, Mead J. 1996 Suction feeding in beaked whales: morphological and experimental evidence. Contrib. Sci. 464.

[52] Aguilar de Soto N, Johnson MP, Madsen PT, Díaz F, Domínguez I, Brito A, Tyack P 2008 Cheetahs of the deep sea: deep foraging sprints in short-finned pilot whales off Tenerife (Canary Islands). J. Anim. Ecol. 77, 936–947. (10.1111/j.1365-2656.2008.01393.x) 18444999

[53] Wings O 2007 A review of gastrolith function with implications for fossil vertebrates and a revised classification. Acta Palaeontol. Pol. 52, 1–16.

[54] Alonso KM et al. 2000 Food habits of the South American sea lion, *Otaria flavescens*, off Patagonia, Argentina. Fish Bull. 98, 250–263.

[55] Shuert CR, Mellish JE 2016 Size, mass, and occurrence of gastroliths in juvenile Steller sea lions (*Eumetopias jubatus*). J. Mammal. 97, 639–643. (10.1093/jmammal/gyv211)

[56] Williams TM et al. 2000 Sink or swim: strategies for cost-efficient diving by marine mammals. Science 288, 133–136. (10.1126/science.288.5463.133) 10753116

[57] Miller PJ, Biuw M, Watanabe YY, Thompson D, Fedak MA 2012 Sink fast and swim harder! Round-trip cost-of-transport for buoyant divers. J. Exp. Biol. 215, 3622–3630. (10.1242/jeb.070128) 23014571

[58] Miller PJO, Johnson MP, Tyack PL, Terray EA 2004 Swimming gaits, passive drag and buoyancy of diving sperm whales *Physeter macrocephalus*. J. Exp. Biol. 207, 1953–1967. (10.1242/jeb.00993) 15107448

[59] Martin Lopez LM, Miller PJO, Aguilar de Soto N, Johnson M 2015 Gait switches in deep-diving beaked whales: biomechanical strategies for long-duration dives. J. Exp. Biol. 218, 1325–1338. (10.1242/jeb.106013) 25954042

[60] Pabst DA, McLellan WA, Rommel SA 2016 How to build a deep diver: the extreme morphology of mesoplodonts. Integr. Comp. Biol. 56, 1337–1348. (10.1093/icb/icw126) 27940620

[61] Velten BP, Dillaman RM, Kinsey ST, McLellan WA, Pabst DA 2013 Novel locomotor muscle design in extreme deep-diving whales. J. Exp. Biol. 216, 1862–1871. (10.1242/jeb.081323) 23393275

[62] Nemoto T, Nasu K 1963 Stones and other aliens in the stomachs of sperm whales in the Bering Sea (*Physeter macrocephalus*). Sci. Rep. Whales Res. Inst. 17, 83–91.

[63] Irvine L, Palacios DM, Urbán J, Mate B 2017 Sperm whale dive behavior characteristics derived from intermediate-duration archival tag data. Ecol. Evol. 7, 7822–7837. (10.1002/ece3.3322) 29043037 PMC5632629

[64] Heezen BC 1957 Whales entangled in deep sea cables. Deep Sea Res. 4, 105–115. (10.1016/0146-6313(56)90040-5)

